# Effect of Pt Nanoparticles on the Optical Gas Sensing Properties of WO_3_ Thin Films

**DOI:** 10.3390/s140711427

**Published:** 2014-06-27

**Authors:** Muhammad U. Qadri, Alex Fabian Diaz Diaz, Michaela Cittadini, Alessandro Martucci, Maria Cinta Pujol, Josep Ferré-Borrull, Eduard Llobet, Magdalena Aguiló, Francesc Díaz

**Affiliations:** 1 Física i Cristal·lografía de Materials i Nanomaterials (FiCMA-FiCNA)-EMaS, Universitat Rovira i Virgili, (URV), Campus Sescelades, c/Marcel·í Domingo s/n, Tarragona 43007, Spain; E-Mails: muhammadusman.qadri@urv.cat (M.U.Q.); magdalena.aguilo@urv.cat (M.A.); f.diaz@urv.cat (F.D.); 2 Dipartimento di Ingegneria Industriale,Universita' di Padova,Via Marzolo 9, Padova 35131, Italy; E-Mails: didialfanet@hotmail.com (A.F.D.D.); michela.cittadini@gmail.com (M.C.); alex.martucci@unipd.it (A.M.); 3 Dept. d'Enginyeria Electronica i Automatica-EMaS, Universitat Rovira i Virgili, (URV), Campus Sescelades, c/Marcel·í Domingo s/n, Tarragona 43007, Spain; E-Mail: josep.ferre@urv.cat; 4 Microsystems and nanotechnologies for chemical analysis (MINOS-EMaS), Universitat Rovira i Virgili, (URV), Campus Sescelades, c/Marcel·í Domingo s/n, Tarragona 43007, Spain; E-Mail: eduard.llobet@urv.cat

**Keywords:** WO_3_ films, optical gas sensing, H_2_, CO, gas, platinum nanoparticles

## Abstract

Thin films of tungsten trioxide were deposited on quartz substrates by RF magnetron sputtering. Different annealing temperatures in the range from 423 to 973 K were used under ambient atmosphere. The influence of the annealing temperature on the structure and optical properties of the resulting WO_3_ thin films were studied. The surface morphology of the films is composed of grains with an average size near 70 nm for the films annealed between 773 and 973 K. Some of the WO_3_ thin films were also coated with Pt nanoparticles of about 45 nm in size. Spectrometric measurements of transmittance were carried out for both types of WO_3_ samples in the wavelength range from 200–900 nm, to determine the effect of the exposure to two different gases namely H_2_ and CO. Films showed fast response and recovery times, in the range of few seconds. The addition of Pt nanoparticles enables reducing the operation temperature to room temperature.

## 1. Introduction

Emissions of different toxic gases such as CO_2_, NO_x_, CO, C_6_H_4_, NH_3_, and CH_4_ from different sources such as industry and automobiles have been polluting our air. These gases can have serious toxic effects on all living beings, especially humans. The damages caused by these gases have increased the demand to control them in the atmosphere. Linked to the green economy and, especially, given the development of new energy systems based on fuel cells, the use of H_2_ gas has been increasing steadily in the last few years. This trend will continue in the years to come. Therefore, it is important to monitor H_2_ in the atmosphere because of its highly explosive nature and low ignition energy [1]. Solid-state gas sensors are very promising for detecting the abovementioned gases, because they represent low cost gas analyzer options [2].

Optical sensors have attracted a lot of interest in the last decades since they allow one to widen the range of operative parameters compared to electrical sensors: in fact, variation in intensity, frequency, polarization and phase of the transmitted/reflected light can be analyzed, and this can in principle improve the device performance by lowering the cross sensitivity among different gases. Some of the advantages of optical gas sensors over conventional conductometric gas sensors are their independence from electromagnetic noise and the possibility of implementing contactless read-out measurements. Eventually they can be implemented in optical fibers and integrated photonic devices allowing fast and easy signal transport and *in situ* measurements with a compact, flexible setup. Moreover they have the capability to transfer the information remotely through an optical fiber network.

Tungsten trioxide, WO_3_, (hereafter TO) has attracted a great deal of interest in the last few years for gas sensing applications. A variety of methods such as radio frequency (RF) sputtering, thermal evaporation, sol-gel, anodization of tungsten films and other chemical synthesis methods have been used to prepare TO thin films for different applications such as electrochromic and optical devices [3–7]. In this paper, we have prepared TO thin films by RF magnetron sputtering. It has been reported in the literature that the fabrication of TO nanostructures by RF magnetron sputtering produces compact thin films with no incorporation of water in their crystal lattices and with a stoichiometry equivalent to the bulk structure [8]. Depending on the method employed for obtaining the films or the deposition conditions during the RF sputtering, TO films may present considerably different structural, optical and electrical properties. TO is a semiconducting material with a large band gap. The optical properties studied in the literature like band gap, spectral transmittance and absorption change of refractive indexes suggest that TO is a good candidate material for future photonic devices and for gasochromic platform based sensing devices [9].

The change in the optical absorbance experienced by oxide materials in the presence of different gases has been a topic of interest in the last years and several mechanisms for such a change have been discussed [10–13]. Being a wide band gap semiconductor, the sensing mechanisms of TO sensors derive from the multiple oxidation states of tungsten. For example, the W^6+^ ions present in TO can be reduced to W^5+^ during exposure to different reducing gases such as H_2_ or CO [14]. Additionally, oxidizing gases are usually chemically adsorbed at the surface of the WO_3_ film removing electrons from its conduction band. The result of these chemical reactions can be visualized through the change of the optical absorbance, serving as a base mechanism for the future optical gas sensor device.

The functional properties of a TO optical gas sensor may be significantly enhanced by lowering the feature size to the nanoscale [15], by structuring film morphology and by improving the crystallographic texture of the grains within the film [16,17]. TO nanostructures (nanorods, nanowires … *etc*.) offer a high surface to volume ratio, which can lead to improved sensitivity towards gaseous components. Recently, TO waveguides activated with metallic nanowires on top have also been reported for hydrogen sensing [18].

The gas sensitivity of metal oxides can be enhanced when they are combined with noble metals such as Pt, Au and Pd [11]. In a previously reported work, Yacoob *et al.* [14] have shown that composite WO_3_ thin films doped with Pt show a significant response at 373 K for optically sensing H_2_. Also, Gaspera *et al.* have shown in [19] that WO_3_ with noble metals such as Au or Pt shows opt-ical sensitivity towards H_2_ and CO at 373 K and at 623 K, respectively. Ando *et al.* [11] also demonstrated the optical gas sensing behaviour of composite WO_3_ films with noble metals (Pt, Au) with significant response at 473 K.

In this paper, RF sputtered films were characterized using atomic force microscopy (AFM), X-ray powder diffraction (XRPD) and μ-Raman spectroscopy. The gas spectral response of such films (deposited onto quartz substrates) upon exposure to H_2_ and CO was studied. The observations were made in the UV-VIS-NIR spectrum (200–900 nm) at room temperature and at 573 K. The aim of this paper is to study the suitability of bare TO films for detecting the presence of gases by absorbance measurements, to evaluate the effect of nanometric roughness and crystalline structure in their optical gas response and to study the effect of the addition of Pt nanoparticles on the optical gas sensing properties of TO films.

## 2. Experimental Section

### 2.1. Preparation of the Films

Thin film structures were prepared on 1 mm-thick, fused quartz substrates. The deposition of TO was performed by RF magnetron reactive sputtering using an ESM100 sputtering system (Edwards, Crawley, West Sussex, UK) with a planar magnetron cathode and a rotatable substrate holder. The sputtering atmosphere consisted of a mixture of Ar–O_2_ and its flow rate was controlled by separate gas flow meters to provide an Ar:O_2_ flow ratio of 1:1. The pressure in the deposition chamber was 5 × 10^−3^ mbar. A tungsten target of 99.99% purity was used. The power was set to 200 W. The substrate was kept at room temperature during film deposition. These conditions of deposition allowed obtaining TO films with a thickness of 550 nm. These films were then annealed in ambient atmosphere for 2 h at different temperatures ranging from 423 to 973 K. The heating and cooling ramps employed in the process of annealing were in both cases 20 K/min.

The deposition of Pt nanoparticles (NPs) onto the TO thin films has been performed using the spin coating technique. Pt NPs, with average diameter of 10 nm, have been synthesized with the polyol method [20]: A solution 80 mM of chloroplatinic acid hydrate (H_2_PtCl_6_) in ethylene glycol was added to a solution of poly(vinyl pyrrolidone) 30 mM and sodium nitrate (NaNO_3_) with a ratio NaNO_3_/H_2_PtCl_6_ = 9 in ethylene glycol, at 433 K. Then, NPs were dispersed in ethanol leading to a 30 mM solution. The Pt NP-deposited samples were heated after the deposition at 473 K during 30 min.

### 2.2. Characterization Techniques

The surface microstructure of the films was examined by AFM (AFM-Pico Plus 2500 from Molecular Imaging, Bid-Service, LLC, Freehold, NJ, USA) operating in the tapping mode and by scanning electron microscopy (QUANTA 600 ESEM, FEI, Dawson Creek Drive, Hillsboro, OR, USA). X-ray powder diffraction (XRPD) measurements were made using a Bruker-AXS D8-Discover diffractometer (Bruker AXS Inc., Madison, WI, USA). The angular range was between 10° and 70° for 2θ, with an angular step of 0.05°, 3 s per step and sample rotation. Cu Kα radiation was obtained with a Cu X-ray tube operated at 40 kV and 40 mA. Also XRPD measurements were done using slow conditions with angular range between 10° and 70° in which the data were collected with an angular step of 0.02°, 16 s per step. With the data obtained from slow conditions, unit cell parameters were refined and the grain size was calculated using the Scherrer formula [21]. Raman spectroscopy measurements were performed at room temperature with an inVia Reflex Raman spectrometer (Renishaw, Gloucestershire, UK) equipped with a 786 nm diode laser. The optical transmittance was measured at room temperature under ambient atmosphere with a Lambda 950 spectrometer (Perkin Elmer, Waltham, MA, USA) in the range from 200–900 nm.

Optical sensor functionality was studied by carrying out optical absorbance measurements over the wavelength range 200–900 nm using a commercial gas flow cell (Harrick, Pleasantville, NY, USA) coupled with a V-570 spectrophotometer (Jasco, Easton, MD, USA). The cell was provided with a heater in order to perform the gas sensing tests up to 673 K. The set-up was the same as fully described in a previous paper [22]. Gas flow was automatically controlled in order to get a continuous, reproducible gas flow inside the measurement chamber at concentrations up to 1 vol·% in dry air. The films were exposed to 1% *v/v* CO and 1% *v/v* H_2_ all balanced in dry air, at a flow rate of 0.4 L/min and at two different operating temperatures, namely room temperature (*i*.*e*., 298 K) and 573 K. The sample area was approximately 1 × 1.5 cm^2^ and the incident spectrophotometer beam was normal to the film surface and covering a 6 × 1 mm^2^ section area. All samples had the same thickness around 550 nm.

## 3. Results and Discussion

### 3.1. X-Ray Powder Diffraction

We have investigated the structural properties of bare TO films by XRPD to identify their crystalline phase, degree of crystallinity and their possible phase modifications due to the different annealing temperatures used. The binary W-O system is rather complex with a large number of phases. Among them, tungsten trioxide can crystallize in many polymorphs with various crystalline structures [9]. According to the literature, the most stable WO_3_ crystallographic phase at room temperature is the monoclinic structure with the space group *P*2_1_/*n*.

**Figure 1.**
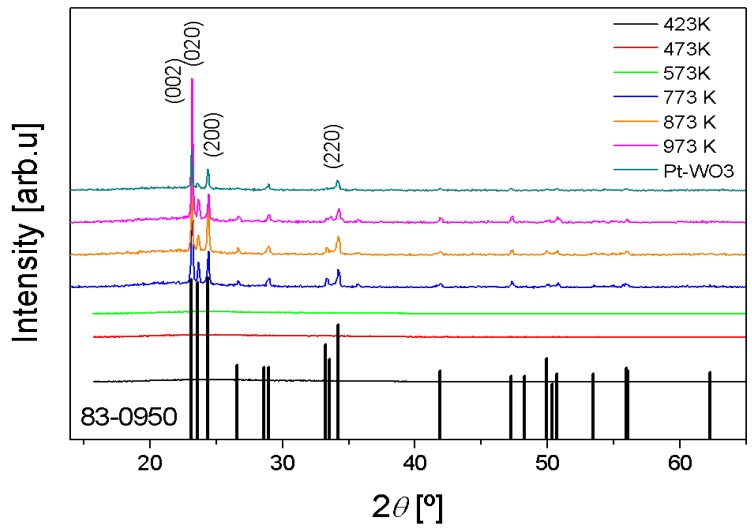
X-ray powder diffraction patterns of the TO films annealed in the range of temperatures from 423 K to 973 K and X-ray powder diffraction pattern of the WO_3_ films annealed at 973 K with Pt nanoparticles.

The obtained XRPD patterns are shown in Figure 1. To determine at which temperature the film starts to crystallize, a broad range of annealing temperatures from 423 K till 973 K was used. As it can be observed, TO films are amorphous when they were annealed in the temperature range from 423 to 573 K. It can also be observed that a crystalline sample is obtained when the annealing temperature is 773 K [16]. An improvement of crystallinity and increase of grain size in TO films when annealed at 773 K has been already reported by several authors [23–25].

This observed crystalline phase belongs to the monoclinic system with the space group *P*2_1_/*n*, in which peaks can be identified using the ICCD 83-0950 pattern with dominant reflection peaks at (002), (020), (200) and (220). The absence of texturing in the film, and the lack of preferential orientation, as expected due to the amorphous nature of the used fused quartz substrate, can also be pointed out. The unit cell parameter of the monoclinic phase has been refined using the Fullprof software [26], in which the Rietveld method is used. The obtained parameters for the annealed sample at 973 K are a = 7.3043(4) Å, b = 7.5430(5) Å, c = 7.6912(4) Å and β = 90.897(1)°.

In our case, for samples annealed at 773 K, 873 K and 973 K, the monoclinic phase is maintained. Also, further increasing of the annealing temperature, usually leads to an improvement in the crystallinity of the samples but this effect is hardly observed in our case. The grain size of crystalline samples was estimated using Scherrer's equation, assuming spherical particles [21] and using the (002) peak. The value of grain size obtained was, in all cases, near 70 nm.

### 3.2. μ-Raman Measurements

**Figure 2.**
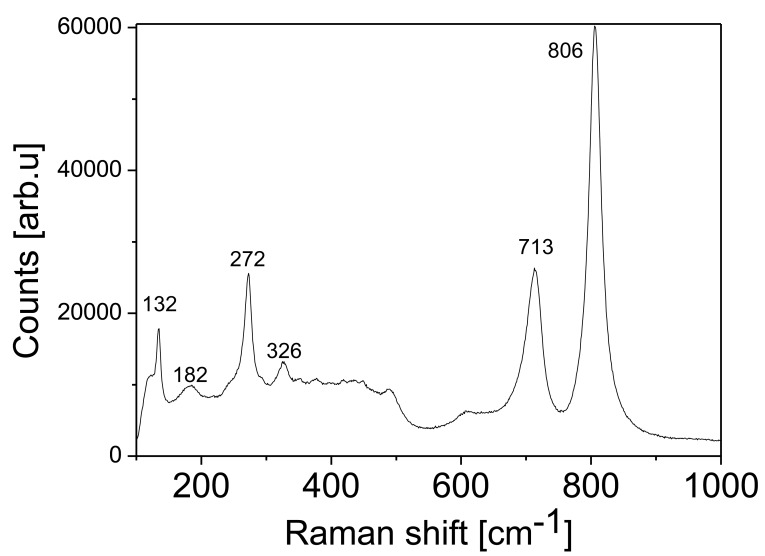
Raman spectra of the TO film annealed at 973 K.

In order to understand in detail the crystalline structure of the films, μ-Raman spectroscopy was performed on the TO thin films annealed at 973 K. The spectrum is shown in Figure 2, in which the phonon peaks can be grouped in a set of two ranges: at high energies 600–900 cm^−1^ and at low energies at 30–400 cm^−1^.

In Table 1 we indicate the values of the phonon modes of tungsten observed with μ-Raman. Raman bands in the 950–1050 cm^−1^ range can be assigned to a symmetric stretching mode of short terminal W = O bonds, ν_s_. The relative intensity of the double W = O bond, typical of non bridging oxygen, is caused by the absorbed water molecules and is frequently seen in sputtered or evaporated films deposited at low temperatures (*i*.*e*., room temperature) [27,28]. These peaks are not observed in our samples, indicating that in our case the presence of adsorbed water is low. The bands in the 750–950 cm^−1^ range are attributed to either the antisymmetric stretch of W-O-W bonds (*i*.*e*., ν[W-O-W]) or the symmetric stretch of (-O-W-O-) bonds (*i*.*e*., ν_s_ [-O-W-O-]) [23].

We have observed Raman bands at 806 and 713 cm^−1^; as described above, these correspond to the stretching vibrations of the bridging oxygen. These bands were also recorded by Daniel *et al.* [27]. The bands at 326 and 272 cm^−1^ can be assigned to δ (O-W-O) vibrations and υ (O-W-O) vibrations. The low phonon bands are attributed to lattice vibrations [24].

### 3.3. Surface Morphological Analysis

The surface and structural morphology of TO thin films were examined by AFM and ESEM. A porous surface with small grain size is recommended for better gas sensitivity [29]. For sensing purposes, polycrystalline and small grains are advantageous because they lead to films with high surface areas for the gas to interact with [30]. On the other hand, a free smooth surface is desirable to obtain better optical response.

**Figure 3.**
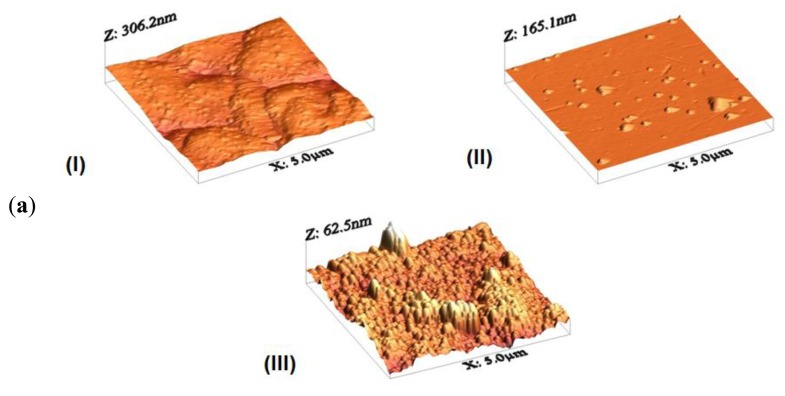

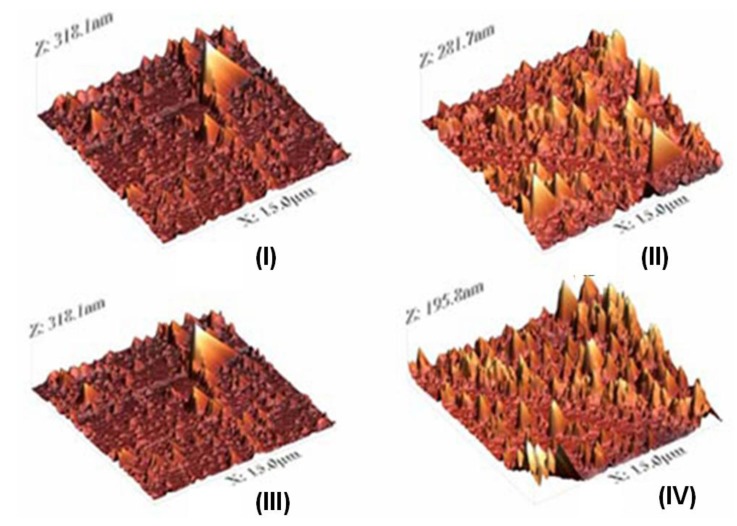
(**a**) AFM micrograph of three different samples annealed at temperatures ranging from 773 K to 973 K. I. annealed at 973 K, II. annealed at 873 K and III. annealed at 773 K; (**b**) AFM micrograph of four different regions of the same sample, annealed at 773 K.

3D AFM images of the crystalline films annealed at temperatures ranging from 773 to 973 K are presented in Figure 3a. The surface is made up of grains and voids with dimensions in the nanometer range. The presence of these voids within the film structure is favorable because they provide direct conduits for gas molecules to flow in from the environment. It can be observed that the grain size of the TO was found to be depending on the annealing temperature [31]. By increasing the annealing temperature, the surface roughness is increased, as indicated by a higher RMS value of the roughness shown in Table 2. This reveals an increase in the size of crystallites with the increase of the annealing temperature. These roughness values are in the range of 40–90 nm. The average grain size found with AFM of three different samples is 67 nm, which agrees well with the value obtained by XRPD. The growth of crystallites, observed to be vertical, could be related to the low rate of atom mobility on the surface of the substrates.

Additionally, different regions of the same sample have been analyzed to evaluate its homogeneity. This can be observed in Figure 3b, which shows the AFM images of four different regions of the sample annealed at 773 K. From this figure it can be observed that samples are quite homogeneous.

**Figure 4.**
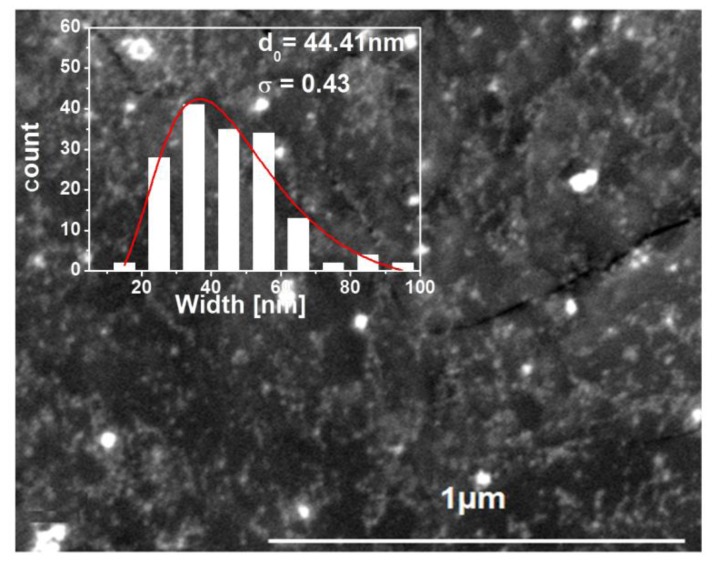
ESEM image of Pt/WO_3_ Pt is visible as bright spots in the image, (inset) the image after size estimation.

The surface morphology of the samples coated with Pt NPs has been studied using ESEM, as observed in Figure 4. These nanoparticles are visible as bright spots in the images. The size of the Pt nanoparticles has been estimated from the ESEM images using the software iTEM. Assuming that the nanoparticles are spherical, the size aspect usually considered is the diameter.

**Figure 5.**
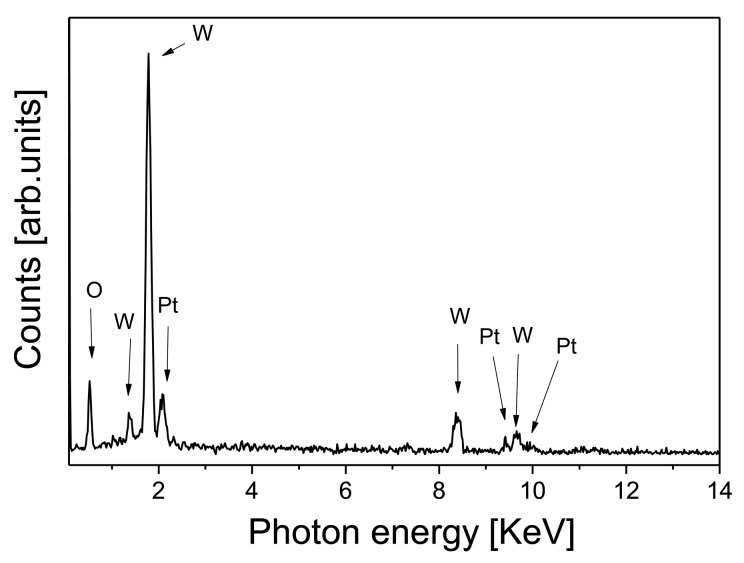
EDS graph of the WO_3_ assisted with Pt.

The particle size distribution is a very important property in powders, and the particle growth processes influence on the size distribution. In [32,33], a description on how the process of nanoparticle formation by nucleation, condensation and particle growth leads to skewed, non-symmetrical size distributions such as the lognormal distribution (the symmetrical normal distribution is usually related to additive effects while the lognormal distribution is related to multiplicative effects [34]). Nanoparticles are expected to yield lognormal size distributions. Indeed, the size histograms of Pt nanoparticles on our WO_3_ thin films are well represented by a lognormal distribution. We fit the log distribution by lognormal function given by:

$$\text{N}\left(\text{d}\right)=\text{A}\exp\left[-{\left(\log\text{d}-\log{\text{d}}_{0}\right)}^{2}/2\sigma^{2}\right]$$

where N(d) denotes the lognormal distribution function being d the determined diameter, A is the amplitude of the mode, d_0_ is the statistical median of the studied diameter, and σ is the dimensionless geometric standard deviation (e^σ^ is the dispersion of the mode). The fits were done directly on the histogram data, and the results are summarized in Figure 4. As derived from Figure 4, we have an average size, d_0_, of 44 nm for the Pt NPs and the first interval of confidence of 68.3% of the size of particles is [28 nm, 67 nm] ([d_0_/e^σ^, d_0_ × e^σ^] [33]. This observed size of the Pt NPs is larger than the one expected by the synthesis process, so this should be due to the fact that we are visualizing nanoparticle agglomerates, instead of single nanoparticles. Energy dispersive X-ray spectroscopy (EDS) has also been performed in order to confirm the presence of Pt NPs. Figure 5 shows the microanalysis graph of the Pt/WO_3_ thin film, and the energy and X-ray emitted by Pt confirms its presence on the doped films.

### 3.4. Optical Characterization

**Figure 6.**
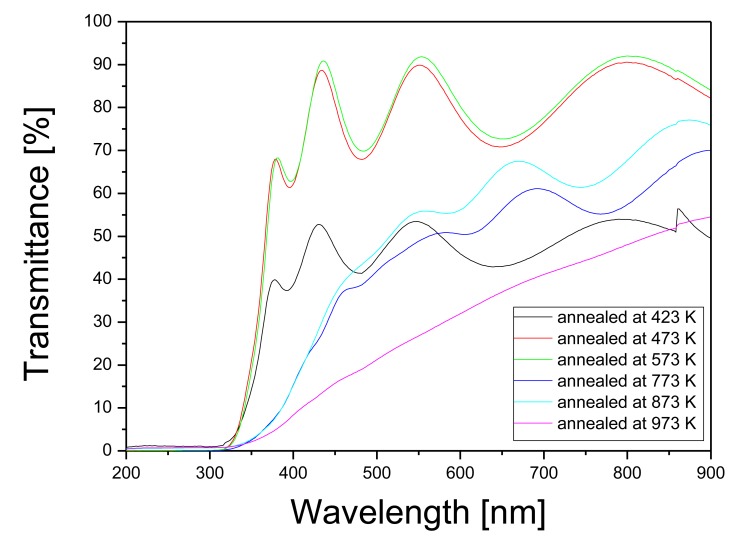
Transmittance measurements of the TO films annealed in the range of temperatures from 423 K to 973 K.

Figure 6 shows the transmittance curves of the TO thin films annealed at different temperatures starting from 423 K to 973 K. Transmittance values in the visible-NIR region range in the interval 44% to 86%. The reflection losses (assuming an ideal flatness and not corrected in Figure 6), calculated using a refractive index value of 2.25 are near 15%.

The maxima and minima in the transmittance curves of these semitransparent films were ascribed to the optical interference that occur in the sputtered TO, due to the multiple reflections of the light when it travels through the thin film. In Figure 6, we plot the transmittance of the samples annealed for 2 h at different temperatures in the range from 423 K to 973 K. The sample annealed at 423 K presents low transmittance and a pattern with important oscillations due to interference in the WO_3_ thin film. With increasing annealing temperature, between 473 K and 573 K, the samples present a similar behavior with an increase of the average transmission, but maintaining the interference fringe pattern. Increasing further the annealing temperature, at 773 K and 873 K, both the average transmission and the amplitude of the oscillations decrease. Finally, at 973 K, a clear decrease of the average transmittance is observed, and the amplitude of the oscillations is completely reduced. The disappearance of these maxima and minima for samples annealed at 973 K agrees with the increase in the roughness of the surface of the film by increasing the annealing temperature. As a result, the scattering increases in high temperature samples and then, the interference process is less probable. Furthermore, the decrease in the transmittance is related to the increase in the roughness (increase in the nanocrystalline grain size) as evidenced from AFM measurements.

Pt NPs don't have visible surface plasmon resonance [35], so they do not present any optical response in the wavelength range studied. Therefore, the transmittance spectra of Pt/WO_3_ are not shown because there are no different features than those of bare WO_3_.

### 3.5. Optical Gas Sensing

The TO thin films have been subjected to optical gas sensing tests by measuring the optical absorption of the thin films under the presence of the two different gases: H_2_ and CO. These two gases do not present optical absorptions in the studied wavelength range. Three different samples annealed at the temperatures of 773 K, 873 K and 973 K were tested. As the samples are homogenous (as observed by AFM), we expect that the measurement results are independent of the position of the optical spot in the surface of the sample.

The bare TO films did not show any detectable gas response at room temperature and at temperatures below 473 K. Thus, the operating temperature was set to 573 K. Higher temperatures generate the appropriate oxygen surface species that will react with the gases, thus promoting an increase in sensitivity [36]. The optical absorption measurements for Pt/WO_3_ were tested at two different temperatures, first at room temperature and then at 423 K.

The absorbance of a given sample exposed to the target gas and the absorbance of the sample exposed to air were compared to define response parameter as Optical Absorbance Change, ΔAbs:

$$\Delta \text{Abs}={\text{Abs}}_{\text{gas}}-{\text{Abs}}_{\text{Air}}$$

**Figure 7.**
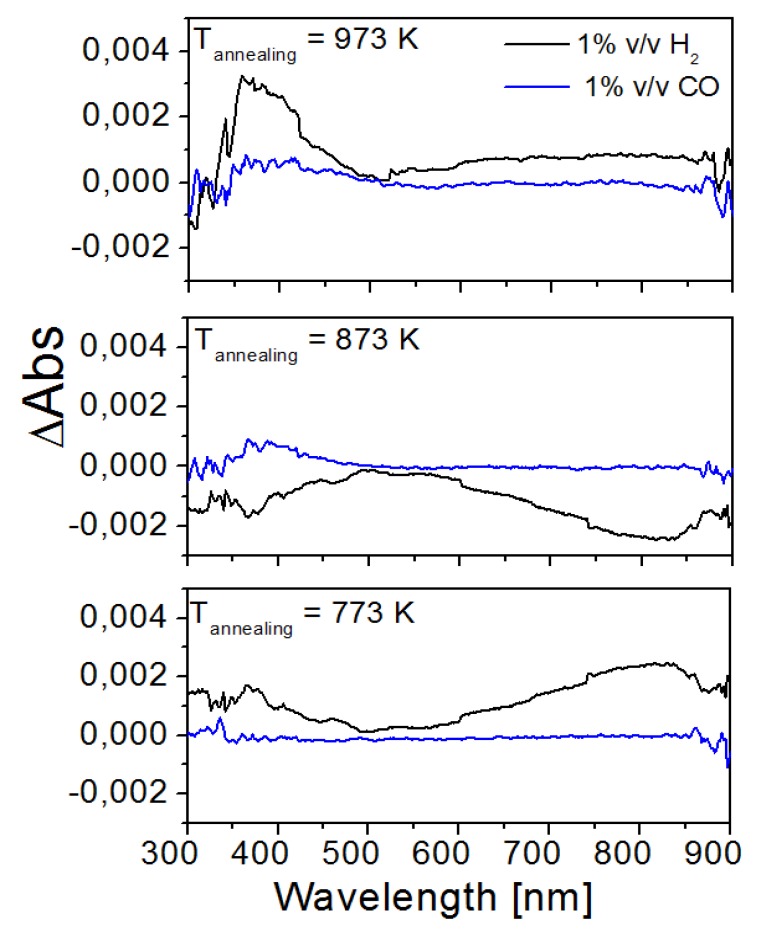
Optical Absorbance Change, ΔAbs = Abs_gas_ − Abs_Air_, curves of samples annealed at 773 K, 873 K and 973 K, for H_2_ and CO at operating temperature 573 K.

The obtained ΔAbs for the four samples tested are shown in Figure 7.

As can be observed in Figure 7, the maximum ΔAbs for each sample occurs in the presence of H_2_. The optical response of the samples to H_2_ increased with the annealing temperature employed in their preparation. However, it can be seen that for each sample, the optimum wavelength for detecting H_2_ varies (near 800 nm, 550 nm and 400 nm for samples annealed at 773 K, 873 K and 973 K, respectively). Bare TO films show some response to H_2_ and are almost irresponsive to CO. It should be pointed out that the TO films discussed here are not decorated with metal NPs and working at a temperature of 573 K. At such operating temperature they show an optical absorption change in the presence of H_2_, which was not observed in pure TiO_2_ at the operating temperatures of 673 K and 773 K [13].

**Figure 8.**
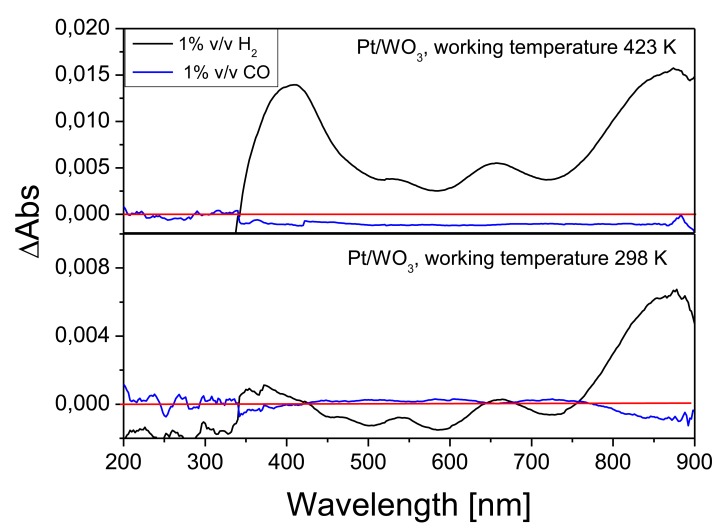
Optical Absorbance Change, ΔAbs = Abs_gas_ − Abs_Air_, curve of Pt assisted WO_3_, for H_2_ and CO at 423 K and room temperature. The red line indicates the zero change in the absorption.

The ΔAbs for Pt/WO_3_ samples, measured at room temperature and at 423 K and in the presence of CO and H_2_ gases (both at a concentration of 1% *v/v*) are shown in Figure 8. In the case of H_2_ measured at 298 K ΔAbs maxima were observed at 850 nm and 660 nm, while at 423 K a further maximum at 400 nm appears. For the CO gas, only a small reduction in absorbance is observed at 423 K in the range from 350 nm to 900 nm. The ΔAbs at room temperature caused by the presence of H_2_ is found to be 0.006 at 850 nm. The ΔAbs caused by CO are minimal as compared to H_2_, for example, it is about 2.54 × 10^−4^ at 760 nm. In a previous publication, Ando *et al.* [11] did not report any response for Pt/WO_3_ composite films after exposing them to H_2_ at room temperature. At a working temperature of 423 K, the maximum response to H_2_ recorded for our Pt/WO_3_ films was close to 0.015 (at 870 nm) and 0.013 (at 408 nm). Unlike for H_2_, when exposed to CO, there were not important changes in the response of Pt/WO_3_ operated at 423 K compared to those measured at RT.

**Figure 9.**
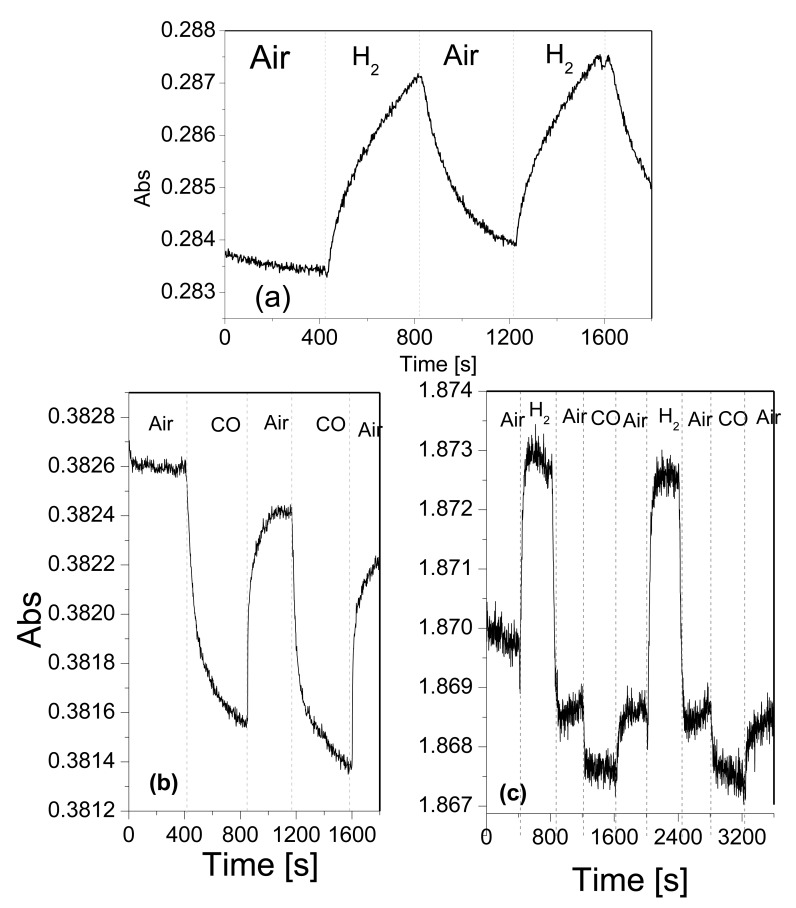
Time resolved absorbance change (ΔAbs ) tests for a Pt/WO_3_ film operated at different temperatures (**a**) ΔAbs recorded at 850 nm for 1% *v/v* H_2_ operating the film at room temperature; (**b**) ΔAbs recorded at 650 nm for the 1% *v/v* CO operating the film at 423 K (**c**) ΔAbs recorded at 340 nm for 1% *v/v* H_2_ and 1% *v/v* CO operating the film at 423 K.

In view of showing that time resolved spectra can be employed as a way to analyze the dynamics of response and recovery cycles. Figure 9 shows the time-resolved measurements of the absorbance for Pt/WO3 films operated at different temperatures. Figure 9a corresponds to the time-resolved response to H_2_ operating the film at room temperature and measuring at 850 nm. Figure 9b corresponds to the response to CO at a working temperature of 423 K and measured at 650 nm. Finally, Figure 9c corresponds to the response to sequential dosing of H_2_ and CO, measured at 423 K and at a wavelength of 350 nm. The choice of the measurement wavelength is based on the optical response observed in Figure 8: If only H_2_ needs to be detected, it can be done at room temperature using the maximum ΔAbs observed at 850 nm. On the other hand, in order to enable CO detection, it is necessary to work at 423 K and choose a wavelength in the range between 350 nm and 900 nm, where a reduction of absorbance is observed. Finally, in order to detect the two gases injected sequentially, the wavelength of 350 nm is chosen because the changes in absorbance for the two gases are of the same order of magnitude and of opposite sign. For Pt coated samples, the absorption change observed for H_2_, at 850 nm is high compared to the other gases tested. This could be foreseen since Pt is known to increase the sensitivity towards H_2_ in metal oxides [22,37,38]. So, the observed optical absorption change should be related to the adsorption of dissociated H^+^ on WO_3_ and the consequent change in optical properties of WO_3_. The Pt NPs are optically inactive in the studied wavelength range and their role is exclusively catalytic. Thus, the dynamic response of H_2_ at 850 nm could be measured at RT (observed in Figure 9a). The response time induced by a step change from air to H_2_ is around 6 min. Also, as reported earlier for other noble metals, the baseline is not fully recovered due to the slow recovery from H_2_ exposure at low operating temperatures [11]. Shanak *et al.* [12] report slow recovery, in the range of 50 min at room temperature, for Pt doped WO_3_ films. In contrast, here, we have comparatively fast response in the range of few minutes at room temperature. Probably the small size (in the range of nanometers) of our Pt NPs explains a higher catalytic activity, even at room temperature. In that case, Pt NPs either dissociate the hydrogen molecule and then hydrogen atoms spill over the metal oxide and diffuse until they reach a reaction site (active oxygen surface species) or promote the transfer of charge carriers between the gas molecule and the metal oxide via the Pt/TO interface (Fermi level control) [39]. Both these mechanisms eventually translate in changes in the optical properties of the material.

The dynamic responses at 650 nm for CO and at 340 nm for H_2_ and CO are plotted in Figure 9b,c, respectively. The response time for CO at 650 nm is about 300 s, but at 340 nm response time is reduced down to 40 s. Dynamic resistance measurements of Fe activated WO_3_ films for CO sensing at 423 K, reported similar response times of about 64 s [40]. The response time for H_2_ is about 55 s at 340 nm. Longer response times of about 200 s have been obtained when using SnO_2_ nanoparticle films as resistive gas sensors for detecting H_2_ at 623 K [41].

Ando *et al.* [11] reported that their sputtered Pt/WO_3_ composite films responded at a temperature of 473 K and found no response at room temperature. Shanak *et al.* [12] reported the colouration with undisclosed H_2_ concentration at room temperature. Both of them reported baseline recovery and slow recovery issues in the range of 50 min. Yaacob [14] also reported high response and recovery time in Pt/WO_3_ samples. The results on the gas sensing properties of Pt/WO_3_ presented here show high stability, and significantly faster recovery at room temperature and at 423 K than in previously reported studies. Table 3 summarizes the details of the responses at the different operating temperatures and different wavelengths employed.

## 4. Conclusions

We have investigated the optical and structural properties of TO thin films grown by RF sputtering with thickness near 550 nm. The annealing conditions control the final crystalline phase or amorphous form of the TO films. The crystalline phase identified using XRPD, of the films annealed at 773 K and above is monoclinic and the films annealed below 773 K are amorphous. The transmittance of the TO films decreased with the increase in the annealing temperature. The increase in the crystallinity causes the optical band-gap to shift to longer wavelengths. The functionality of these thin films has been analyzed under the presence of two different gases, namely, H_2_ and CO. These bare TO films show a minor optical response to H_2_. The TO films coated with Pt NPs show optical response at RT (which has not been reported in previously published works) and also at 423 K. The response time of several seconds at the operating temperature of 423 K is found to be in agreement with the literature. The best responses to the gases tested were obtained for the Pt containing samples operating at 423 K. This material was found to be highly sensitive to hydrogen.

## Figures and Tables

**Table 1.** Raman modes for WO_3_ film prepared by RF magnetron sputtering.

**Raman Shift [cm^−1^]**	**Assignment**	**References**
806	W-O-W	[24,25]
713	W-O-W	[25]
326	(WO_3_)	[4]
272	((O-W-O) δ (O-W-O)	[25]
182	W-W	[25]
132	-	[25]

**Table 2.** Surface morphological analysis of different samples annealed on different temperatures using AFM.

**Sample**	**I**	**II**	**III**
**Annealing Temperature [K]**	973	873	773
**RMS [nm]**	90.66	44.76	42.17
**Average Roughness [nm]**	85.01	43.23	40.32
**Average Height [nm]**	96.44	53.55	49.20

**Table 3.** Table summarizing gas responses and response times.

**Samples**	**H_2_**		**CO**	
	
	**Δabs**	**τ**	**Δabs**	**τ**
WO_3_-Pt 300 K	0.006 (850 nm)	360 s (850 nm)	2.54 × 10^−4^ (760 nm)	-
WO_3_-Pt 423 K	0.015 (870 nm) and 0.013 (408 nm)	40–45 s (650 nm)/ 55 s (340 nm)	∼2.54 × 10^−4^ (650 nm)	300 s (650 nm)/ 40 s (340 nm)
